# Does Every Strain of *Pseudomonas aeruginosa* Attack the Same? Results of a Study of the Prevalence of Virulence Factors of Strains Obtained from Different Animal Species in Northeastern Poland

**DOI:** 10.3390/pathogens13110979

**Published:** 2024-11-08

**Authors:** Paweł Foksiński, Alicja Blank, Edyta Kaczorek-Łukowska, Joanna Małaczewska, Małgorzata Wróbel, Ewelina A. Wójcik, Patrycja Sowińska, Nina Pietrzyk, Rafał Matusiak, Roman Wójcik

**Affiliations:** 1 Department of Microbiology and Clinical Immunology, Faculty of Veterinary Medicine, University of Warmia and Mazury in Olsztyn, Oczapowskiego 13, 10-719 Olsztyn, Poland; ala.blank@gmail.com (A.B.); edyta.kaczorek@uwm.edu.pl (E.K.-Ł.); joanna.malaczewska@uwm.edu.pl (J.M.); malgorzata.wrobel@uwm.edu.pl (M.W.); brandy@uwm.edu.pl (R.W.); 2 Proteon Pharmaceuticals, Tylna 3a, 90-364 Łódź, Poland; ewojcik@proteonpharma.com (E.A.W.); psowinska@proteonpharma.com (P.S.); npietrzyk@proteonpharma.com (N.P.); rmatusiak@proteonpharma.com (R.M.)

**Keywords:** *Pseudomonas aeruginosa*, animal samples, biofilm, ERIC-PCR, genotyping, virulence genes

## Abstract

Background: *Pseudomonas aeruginosa* is a pathogen that causes infections in animals and humans, with veterinary implications including ear infections in dogs, respiratory diseases in cats, and mastitis in ruminants. In humans, it causes severe hospital-acquired infections, particularly in immunosuppressed patients. This study aimed to identify and assess the prevalence of specific virulence factors in *Pseudomonas aeruginosa* isolates. Methods: We analyzed 98 *Pseudomonas aeruginosa* isolates from various animal samples (dogs, cats, ruminants, fowl) from northeastern Poland in 2019–2022 for virulence-related genes (toxA, exoU, exoT, exoS, lasB, plcN, plcH, pldA, aprA, gacA, algD, pelA, endA, and oprF) by PCR and assessed biofilm formation at 48 and 72 h. Genomic diversity was assessed by ERIC-PCR. Results: The obtained results showed that all strains harbored the *pel*A gene (100%), while the lowest prevalence was found for *pld*A (24%) and *exo*U (36%). Regardless of the animal species, strong biofilm forming ability was prevalent among the strains after both 48 h (75%) and 72 h (74%). We obtained as many as 87 different genotyping profiles, where the dominant one was profile ERIC-48, observed in four strains. Conclusions: No correlation was found between presence or absence of determined genes and the nature of infection. Similarly, no correlation was found between biofilm-forming genes and biofilm strength. The high genetic diversity indicates challenges for effective prevention, emphasizing the need for ongoing monitoring and research.

## 1. Introduction

*Pseudomonas aeruginosa* (*P. aeruginosa*) is an important bacterium in both human and animal medicine. This is related to its ability to cause a variety of infections in both fully immunocompetent and immunocompromised organisms. Due to its numerous defense mechanisms, genetic elasticity and high antibiotic resistance, this pathogen represents a constant challenge in modern medicine [1].

In humans, this bacterium is usually associated with ears, lungs, urinary tract, eyes and skin infections (mainly wounds). In addition, in immunocompromised individuals as a result of primary diseases or long-term antibiotic therapy, they can cause intestinal infections that can lead to sepsis [2]. It is assumed that the etiological agent of as many as 7.1–7.3% of healthcare-associated infections is *P. aeruginosa* [3], where, over the years, the frequency of this bacterium in disease entities has oscillated: with hospital-acquired pneumonia (15.6–21.8%), pneumonia associated with mechanical ventilation (19.4–25.9%), cystic fibrosis (60–70%), urinary tract infections (7–17%), corneal infections (6.8–55%), wound infections including those due to burns (10–57%), and infections of immunosuppressed patients excluding burns (8–25%) [4].

In animals, this pathogen is associated with different animal species. In the case of companion animals (mainly cats and dogs), it is most commonly associated with clinical signs of otitis externa, lower urinary tract infections, pneumonia [5], and skin and wound infections [6]. When it comes to livestock, in ruminants, it is one of the etiological factors causing mastitis [7]. In the poultry industry, *P. aeruginosa* is the cause of many infections among avian species, leading to increased mortality in all age groups, and it can also cause embryonic death in contaminated hatcheries [8]. In the case of reptiles, it represents a normal part of the bacterial flora of their oral cavity and gastrointestinal tract, which may pose a risk of transmission to humans, through close contact between captive individuals and their owners [9]. Unfortunately, there are very few data on the incidence of *P. aeruginosa* infections in veterinary medicine. In dogs, Hattab et al. reported that among the clinical cases they considered, 8% were due to *P. aeruginosa,* accounting for 25% of otitis media infections, 10% of skin infections and 1.6% of urinary tract infections [10]. This bacterium is believed to be the most common cause of otitis in dogs, and its prevalence is estimated at 25–41% for this infection [11]. Other data show that the prevalence of *Pseudomonas* spp. infection is 16% for dogs and 10% for cats, of which *P. aeruginosa* species accounted for 92% and 72%, respectively [12]. In cattle, the incidence of *Pseuddomonas aeruginosa* was recorded by Badawy et al. at 26.4% [13]. In poultry, the incidence of *P. aeruginosa* can range from 8.7% to 39.78%, and in dead embryos, it can reach up to 52% [14].

*P. aeruginosa* is resistant to many antibiotics, which can lead to an overuse of drugs with non-targeted therapy and result in an increase in residual ineffective substances in the environment, causing an increase in the phenomenon of bacterial drug resistance [15]. The basis for the spread of this phenomenon is horizontal gene transfer and the high adaptability of the genome of this bacterium, which also results in a variety of pathogeneses of infection and allows them to survive in adverse environmental conditions [16,17].

Data on antibiotic resistance among strains obtained from humans highlight the significant variability in resistance rates, with the highest resistance observed for fluoroquinolones and cephalosporins, such as gatifloxacin (87.2%) and ceftriaxone (80.9%). For carbenicillin, the level was comparable to them (80.5%), while a decrease in resistance was observed for other drugs: cefepime (42.7%), ciprofloxacin (34.3%), imipenem (30.5%), ceftazidime (29.2%) gentamicin (26%) piperacillin/tazobactam (24.2–90%), tigecycline (5%), polymyxin B (3.1%) and colistin (2.4–3.1%) [18,19].

Based on recent data, among dogs and cats, depending on the region, resistance of *P. aeruginosa* to antibiotics is recorded at different levels: amoxicillin with clavulanic acid (100%), amikacin (0–55%), aztreonam (0–59%), azithromycin (41.37–48%), ceftazidime (0–53.44%), ciprofloxacin (8.7–86.2%), clindamycin (100%), colistin (2.6–54%), enrofloxacin (4–68%), cefepime (0–64%), florfenicol (100%), gentamicin (0–62%), imipenem (0–78%), kanamycin (95%), marbofloxacin (21–32%), meropenem (3.4–74%), polymyxin B (92–98%), tetracycline (89.7%), tobramycin (6.8–91%), piperacillin–tazobactam (0–74%), and trimethoprim/sulfamethoxazole (92.3%) [20,21,22,23]. In ruminants, resistance was observed at the following levels: sulfamethoxazole (100%), imipenem (14.8–72.2%), cefepime (72.2%), ceftazidime (38.9%), ciprofloxacin (59.3%), piperacillin (77.8%), piperacillin–tazobactam (68.8%), genatmycin (50–63.3%), and colistin (0%) [13,24]. In contrast, in poultry, there was complete resistance to amoxicillin, amoxicillin with clavulanic acid, ampicillin, doxycycline, erythromycin, nalidixic acid, sulfamethazine, and tetracycline; less resistance to levofloxacin (0–81.25%), enrofloxacin (59.375%), and danofloxacin (46.875%); and sensitivity to ciprofloxacin, gentamicin, and norfloxacin [14].

In addition, treatment of the infection is hindered by the bacteria’s ability to form a biofilm, which limits the penetration of antibiotics through its structure and allows the formation of persister cells that are able to survive treatment with many times higher concentrations of the antibiotic due to their planktonic form, and causes recurrence of the infection once the treatment has ended [25].

A biofilm structure is a concentration of bacterial cells suspended in an extracellular matrix (EPS) that they produce themselves from polysaccharides, proteins, extracellular DNA (eDNA) and lipids. The biofilm structure consists of approximately 10% bacteria and 90% EPS [26]. The formation of this structure is influenced by genetic factors, such as the presence of genes encoding surface factors like pili, flagella, alginate, lectins, lipopolysacharyde responsible for the bacterial adherence to the surface and colonization of the host [27], and their expression depending on adverse conditions (temperature variation, nutrient availability, pH, host conditions and others) [28].

These bacteria are capable of forming a biofilm both in living organisms and on non-organic surfaces, which is usually the result of environmental stress affecting the bacteria, which react as a defensive response. One of the side effects of the formation of this structure is the increased resistance of these bacteria to antibiotics [29].

The difficulty of treatment is also influenced by the presence of virulence factors other than those related to biofilm formation, such as the production of cytotoxins, elastases, hemolysins, neuramidases and proteases, which also determine the virulence of pathogenic strains, leading to aggravation of the infection [27].

The pathogenesis of *P. aeruginosa* infection is a multifactorial process. Whether the infection develops into an acute or chronic infection depends on the severity of the expression of extracellular factors. Those associated with the cell itself, largely responsible for biofilm production, cause chronic disease development, while the increased expression of genes encoding extracellular factors causes the development of acute disease development [27]. Which genes undergo increased expression is regulated by quorum sensing (QS), a type of bacterial communication mediated by signaling molecules (acyl-homoserine lactones, AHLs) in the biofilm structure [30]. When microbial abundance reaches a high enough density and the concentration of autoinducers (AHLs) reaches threshold values, gene expression is altered. QS regulates the expression of genes responsible for biofilm formation, bacteriocin production and virulence [31].

Considering the slow progress in the development and implementation of new antimicrobial agents, there is a need to develop effective treatment alternatives for infections with resistant pathogens, which include *P. aeruginosa* [32]. New therapies for the treatment of these infections include quorum sensing inhibitors, immunotherapy, iron chelators, vaccines, herbal medicine, and phage therapy [33].

The occurrence of virulence factors varies depending on the host, geographic latitude, or type of infection site [34]. Monitoring such mechanisms allows the development of targeted preventive actions, which is important because of the heavy character of infection treatment. Therefore, the purpose of this study was to compare the prevalence of genes encoding chosen virulence factors and phenotypically determine biofilm-forming ability among *P. aeruginosa* strains isolated from different animal species in north-eastern Poland, which were characterized in terms of genomic relatedness.

## 2. Materials and Methods

### 2.1. Collection of Strains

A total of 98 *P. aeruginosa* strains were collected and isolated from clinical samples as a primary cause of infection in companion and livestock animals (dogs, cats, ruminants, and fowl) from the northeast of Poland between 2017 and 2021. Full details of the isolated strains can be found in Table S1 in the Supplementary Materials.

### 2.2. Isolation and Identification

Isolation and identification were carried out according to the type of sample received. In the case of swabs, samples were pre-inoculated in TSB (Tryptic Soy Broth) broth (Oxoid, Basingstoke, Great Britain) for 24 h under aerobic conditions at 37 °C. After this time, samples were inoculated onto Columbia Agar, TSA (Tryptic Soy Agar) and Cetrimide Agar (all media were from Oxoid, Basingstoke, Great Britain) and were then incubated for 48 h under aerobic conditions at 37 °C. Liquid samples were directly seeded onto the above-mentioned solid media and also incubated under the same conditions. The grown isolates were subjected to microbiological analysis, which included the evaluation of the morphology of bacterial colonies, pigment production, smell, gram staining, motility and selected biochemical tests (oxidase, catalase, urease, indole and citrate). The final identification of *P. aeruginosa* was confirmed by PCR reaction.

### 2.3. Extraction of DNA

The genomic DNA was extracted from each of 98 strains after overnight culture on LB (Luria Broth) agar using the Wizard^®^ SV Genomic DNA Purification System from Promega (Madison, WI, USA) according to the manufacture’s procedure.

### 2.4. Pseudomonas Genus Identification PCR

Primers for the detection of *Pseudomonas* genus were designed after a comparative analysis of the ribosomal protein S10 coding region. The sequences of these coding DNA fragments were extracted by local BLASTn from all complete *Pseudomonas* genomes (8582 genomes), which were downloaded from the *Pseudomonas* database (https://www.pseudomonas.com/, online access 2 July 2020) [35]. Extracted sequences were aligned by MAFFT algorithm [36] to detect the conservative regions for the *Pseudomonas* genus. The conservative regions were analyzed by BLASTn [37] for the detection of regions that are strictly specific for *Pseudomonas* genus. The specific regions were screened manually with the support of SnapGene Viewer 4.3.117 (www.snapgene.com, accessed on 29 August 2022). Primers’ thermodynamic properties were analyzed using Oligo Calc [38] and Primer-Dimer [39]. In silico PCR showed a sensitivity of 99.45% and 100% specific designed primers.

### 2.5. Pseudomonas Identification—PCR Amplification

All isolates were checked in terms of genus and species. Amplification was performed in the reaction mixture with a final volume of 50 µL, containing primers at a concentration of 10 µM, PCR-grade water, 2× Color Taq PCR Master Mix (EURx, Gdańsk, Poland): 2× concentrated, ready-to-use mixture for PCR reactions containing PCR reaction buffer, MgCl_2_, dNTPs, Taq recombinant DNA polymerase, and 1 µL of template DNA. For genus verification, primers Ps_30S_S10_F (CCCAGGAAATCGTGGAAACCG) and Ps_30S_S10_R (CGCCCATTTCAGAGCGTTACAC) were used, while for species verification PA-SS_F (GGGGGATCTTCGGACCTCA) and PA-SS_R (TCCTTAGAGTGCCCACCCG) were used. The reaction was performed using a Nexus Gradient thermocycler (Eppendorf, Hamburg, Germany) under the following conditions: (i): 94 °C, 2 min—initial denaturation, 30 cycles of (ii): 94 °C, 15 s—denaturation, (iii): 65 °C—genus/64 °C—species, 1 min—annealing and elongation, (iv): 72 °C, 7 min—final elongation, 4 °C—cooling. PCR amplification of specific DNA fragments identifying the genus (286 bp) or species (956 bp) were performed with electrophoresis-visualizing PCR products. The separation was carried out on 1.5% agarose gel in 1× TAE buffer for 1 h at a voltage of 32 V/cm gel length.

### 2.6. Detection of Virulence Factors by PCR Reaction

Polymerase chain reaction (PCR) was performed for the detection of the presence of fourteen virulence-related genes: *tox*A, *exo*U, *exo*T, *exo*S, *las*B, *plc*N, *plc*H, *pld*A, *apr*A, *gac*A, *alg*D, *pel*A, *end*A, and *opr*F. Primer sequences, product sizes, and annealing temperatures are summarized in Table S2 in the Supplementary Materials [10,40,41,42,43,44,45,46,47,48,49]. All primers were synthesized using Genomed S.A. (Warsaw, Poland). Amplification reactions and visualization were carried out in the same way as in the previously published article [50].

### 2.7. Biofilm Formation

Biofilm capacity testing was performed according to Ebrahimi A. et al. 2013 [51] with minor modifications. *P. aeruginosa* isolates were grown in TSB broth for 24 h and then standardized to obtain equal amounts of bacteria in each sample (5 × 10^8^ cells/mL) with 1% glucose and TSB solution. Measures of 200 µL of bacterial solution with 1% glucose (1:40 dilution) were transferred to microtiter plates and incubated at 37 °C. After 48 and 72 h, the plates were stained with crystal violet, and the results were read using an ELISA plate reader (Sunrise absorbance reader, Tecan, Austria) and interpreted according to Stephanovic S. et al. (2007) [52].

### 2.8. ERIC-PCR Genotyping

All 98 strains were exposed to ERIC-PCR (Enterobacterial Repetitive Intergenic Consensus Polymerase Chain Reaction) to check their genomic relatedness. Amplification was performed in the reaction mixture with a final volume of 50 µL, containing primers at concentrations of 0.2 µM with sequences ERIC-1′: CACTTAGGGGTCCTCGAATGTA and ERIC-2: AAGTAAGTGACTGGGGTGAGCG, 5 mM MgCl_2_ (BLIRT, Gdańsk, Poland), 200 µM deoxynucleoside triphosphate dNTP (BLIRT, Gdańsk, Poland), 2 U PWO polymerase (BLIRT, Gdańsk), 5 µL 10× SHARK buffer (BLIRT, Gdańsk, Poland) and 5 µL of template DNA. The PCR reactions were performed as follows: (i) 5 min at 95 °C—initial denaturation, 35 cycles of (ii): 45 s at 92 °C, (iii) 1 min at 45 °C—annealing and elongation at 70 °C for 10 min and final elongation at 72 °C for 20 min, cooling: 4 °C.

The separation of ERIC-PCR products was carried out on 1% agarose gel in 1 × TAE buffer for 3 h at a voltage of 32 V/cm gel length.

All gels were analyzed using the BioNumerics version 5.0 software package (Applied Maths, Sint-Martens-Latem, Belgium). Dendrograms for ERIC-PCR gels included in the Figure S1 of the Supplementary Materials were generated using the Dice similarity coefficient and UPGMA algorithm (unweighted pair group method with arithmetic mean).

## 3. Results

### 3.1. Strains—Place of Isolation and Host

Among 98 *P. aeruginosa* strains isolated from companion and livestock animals from the northeast of Poland between 2017 and 2021, biological samples from which the strains were isolated were collected from various areas. The majority of strains from dogs were isolated from cases of otitis, with the respiratory system and skin infections being the next most common sources. In cats, almost all of *P. aeruginosa* strains were isolated from the nasal cavity. In the case of fowl and ruminants, the strains were isolated from the goiter, cloaca and milk samples, respectively (Table 1).

### 3.2. Detection of Virulence Genes in General

**Figure 1:**
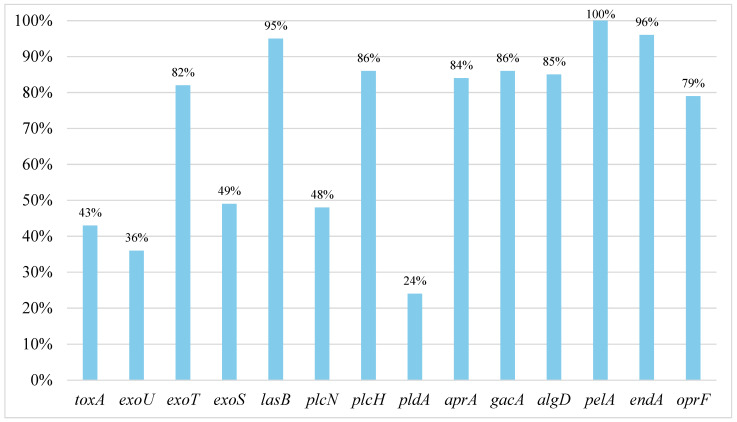
Percentage frequency of virulence genes (*tox*A, *exo*U, *exo*T, *exo*S, *las*B, *plc*N, *plc*H, *pld*A, *apr*A, *gac*A, *alg*D, *pel*A, *end*A, *opr*F) among the analyzed *P. aeruginosa* strains.

All of the fourteen virulence genes we determined were confirmed among the strains analyzed. The most common were *pel*A (100%), *end*A (96%) and *las*B (95%). The least frequently occurring gene was *pld*A (24%). The remaining genes occurred at different frequencies, ranging from 36 to 86%. Detailed information on the frequency of the genes we analyzed can be found in Figure 1.

### 3.3. Detection of Virulence Genes in Individual Animal Species

The most common genes besides *pel*A (100%) among dogs were *end*A (95%) and *las*B (92%). In the case of cats, the most common genes were *las*B (100%), *plc*H (94%), *gac*A (94%), *end*A (94%) and *opr*F (94%). Among ruminants, the dominant were *las*B (100%), *gac*A (100%), *alg*D (100%) and *end*A (100%). In poultry, the most common were *las*B (100%) and *end*A (100%). There was no correlation between the presence or absence of determined genes and the nature of the infections (Table 2).

### 3.4. Biofilm Formation in General

The results from examination of the ability of the strains to form biofilm after 48 and 72 h are presented in Table 3. At the two analyzed times, the results were similar. Strong biofilm producers were predominant (74–75%), moderate producers represented 18–19%, while a weak biofilm was produced by only 4% of the strains. Among all the strains analyzed, only one was not confirmed to have the ability to produce biofilm (Table 3).

### 3.5. Biofilm Formation in Individual Species

**Figure 2:**
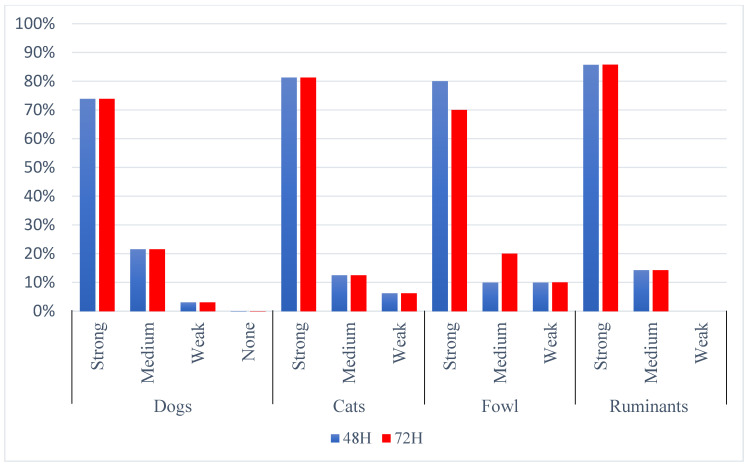
Percentage of strong, medium and weak biofilm producers by species after 48 and 72 h.

In individual host species, the distribution of bacteria capable of producing biofilm presented a similar picture to the overall analysis, and no differences were noted between the strength of biofilm production capacity and the animal species. Among all the hosts analyzed, strains with a strong biofilm predominated (74 to 86%). Most weak producers were observed in fowl, cats and dogs. In cattle, no such strains were found. The strain with no biofilm production was from a dog (Figure 2).

### 3.6. Occurrence of Biofilm-Related Genes and the Ability to Produce Biofilms Phenotypically

The analyzed strains were checked for the presence of virulence genes associated with biofilm formation (*pel*A, *alg*D, *end*A and *opr*F). Analysis showed that the *pel*A gene was present in all strains with no difference in the classification of producers by biofilm strength. The *end*A gene was dominant in strong and medium producers (above 90%) and *alg*D in weak producers (100%). The *opr*F gene was present in all analyzed strains capable of biofilm production at (72–80%) (Table 4).

### 3.7. Genotyping

For all 98 isolates, ERIC genotyping was performed, and for these samples, profiles of bands were obtained (Figure S1 in the Supplementary Materials). Using this method, 87 patterns were highlighted. The dominant one was profile ERIC-48, observed in four strains. A summary of obtained ERIC profiles for *P. aeruginosa* isolates is presented in Table S1 in the Supplementary Materials.

## 4. Discussion

Infections caused by *P. aeruginosa* in both humans and animals represent an ever-growing problem. This is due to the increasing multidrug resistance to the active substances used in both human and veterinary treatment accompanied by an increase in their virulence (especially biofilm production) [31,53].

The virulence factors that were determined in our study are responsible, among others, for the ability to damage cell membranes, deactivate elements of the immune system, or increased adherence to surfaces that allow bacteria to survive in adverse conditions. Most of the analyzed strains show the presence of numerous virulence genes, which indicates the diversity of the pathogenesis of the infections caused. However, no correlation was observed between the presence or absence of genes and host species. It is important to note that the mere presence of a gene in a bacterial genome does not necessarily correlate with an increase in virulence, as genes do not always function independently. Conversely, the absence of a specific gene may not result in reduced virulence, given the potential existence of a functionally analogous gene with a distinct sequence that could compensate for the missing function [54]. Therefore, experimental infections in an animal model are required to assess the virulence of the strains examined in this study. Genetic variability between different bacterial strains associated with various adaptive mechanisms allows pathogens to colonize a range of host animal species [55]. Bacteria, through evolution, have developed diverse mechanisms that allow them to grow and develop under different conditions [56].

*P. aeruginosa* has six secretion systems (T1SS–T6SS), secreting a wide range of toxins and hydrolytic enzymes that allow it to modulate the immune system of the host. The first and fifth systems (T1SS and T5SS) are the least complex extracellular secretion pathways, with the first responsible for, among others, the secretion of *apr*A gene expression products [57]. The second (T2SS) is the most versatile system, secreting a wide range of proteins, and is responsible for secretion of gene expression products such as *tox*A, *las*B, *plc*H, *plc*N [58]. The most important secretion system for bacteria is T3SS, as it secretes the exotoxins *exo*T, *exo*S, and *exo*U, among others, responsible for inactivating and destroying host immune cells [57,59]. The sixth system (T6SS) secretes, for example, phospholipase D (*pld*A) to destroy the host’s bacterial flora [43]. Among the strains we collected, as many as 84% contained genes whose expression products are secreted by T1SS, 100% by T2SS, 92% by T3SS and 24% by T6SS. We have not identified all of the genes secreted by these systems, but given their commonness, it can be assumed that even if the expression of these genes does not currently occur, there is nothing to prevent it from doing so, which could pose a threat in the future.

Exotoxin A encoded by *tox*A gene is an extracellular enzyme produced by 43% of our isolates. It is responsible for transferring the adenosine diphosphate–ribosyl grouping from nicotinamide adenine dinucleotide to elongation factor 2 in host cells, resulting in its inactivation and inhibition of cell protein biosynthesis. It also exerts a direct cytopathic effect and interferes with the immune response [60]. Alabdali et al. (reference) found that the frequency of the gene responsible for the production of exotoxin A among *P. aeruginosa* strains was 35%, and they suggested that this correlated with increased antibiotic resistance [61]. Since we have a higher prevalence of this gene, this may indicate a higher level of antibiotic resistance among the strains we analyzed. Nevertheless, at the moment, we have not carried out such studies, so these are only our predictions. Therefore, further research should be conducted.

Exotoxin U was determined by us in 36% of the strains, which agrees with the observations of Deruelle et al. 2021, who found that the frequency of the *exo*U gene encoding this toxin oscillates between 28 and 48% of *P. aeruginosa* strains [62]. Due to its phospholipase A2-like activity, it shows the ability to rapidly lyse mammal cells, making it the most virulent protein secreted by T3SS [63]. Exotoxin U plays an important role in early stages of acute lung infections. It shows affinity for phagocytic cells, causing damage to them, allowing bacteria to multiply in tissues and develop severe disease [64]. Diaz et al. (2010) suggested that *exo*U does not directly cause alveolar cell damage, and that this is likely a secondary effect of neutrophil degradation and the release of inflammatory mediators. They also noted that *exo*U+ strains mostly contain the *exo*T gene and lack the *exo*S gene, which we also observed in our study [65]. About 40% of our strains (*n* = 10) taken from the upper respiratory tract contained the *exo*U gene, which suggests that if treatment is delayed and the infection moved to the lower respiratory tract, it could mean the development of severe lung infections in the animals. Deruelle et al. (2021) also suggested that *exo*U-positive strains often correlated with increased multidrug resistance [62], so we believe that rapid implementation of targeted treatment of respiratory tract infections caused by *P. aeruginosa* is critical.

The frequency of the *exo*S gene in our samples is 49%, while that of *exo*T is 82%. They are bifunctional toxins that interfere with phagocytosis activity and have the ability to destroy the cytoskeleton through expression of the Rho GAP domain [66]. Sarges et al. (2020) showed that *exo*S+/*exo*U- virulootypes are indicative of a chronic form of infection associated with a worse clinical condition than the *exo*S+/*exo*U+ virulotype in human cystic fibrosis. They also noted higher gene frequencies than those determined by us for *exo*U (63.2%), *exo*S (97.9%), *exo*T (95.9%) [67]. In our study, the *exo*S+/*exo*U- virulotype was present among 36 of the strains, where 10 of them were from upper respiratory tract infections, while the *exo*S+/*exo*U+ virulotype was present among 12 of the strains and only 1 was present in upper respiratory tract infections. We suspect that in the future, chronic upper respiratory tract infections involving increased tissue damage in animals may be observed more frequently in our region, likely due to the higher prevalence of the exoS+/exoU- virulotype not only among strains obtained from respiratory tract infections but also from other sites of infection, so the mechanism of this phenomenon should be closely monitored.

The *las*B gene was determined in 95% of our strains. It conditions the secretion of zinc metalloprotease, which destroys host tissues and cells rich in structural proteins such as collagen, non-collagenous proteins and elastin [42]. In addition to its proteolytic activity, the product of *las*B gene expression exhibits immunomodulatory activity in the host, affecting the immune system’s response. It also plays a role for the bacteria itself, stimulating the intracellular pathway responsible for the growth of biofilm structure [68]. In 2020, Wei et al. labeled it at a similar level of 100% [69]. This indicates that the expression products of this gene may play a significant role in the pathogenesis of *P. aeruginosa* infection in our region, both accounting for the ability of the strains we studied to produce biofilm and the intensity of the infections they cause. In 2011, Cathcart et al. showed that the use of inhibitors for this gene combined with antibiotic therapy eliminated the biofilm, so we think that because *las*B was so prevalent in our study, it is worth considering using it to treat infections in our region [68].

The least frequent gene among the genes we analyzed was *pld*A, encoding phospholipase D, present in 24% of samples. These results are consistent with the observations of Luo et al. (2019), where the frequency of this gene was 18.5% [70]. This gene may be responsible for effective colonization of cell membranes by causing changes in phosphatidylinositol (3,4,5)-trisphosphate, a phospholipid that resides on the plasma membrane and in actin in host cells [71]. Nevertheless, its low prevalence among the strains we analyzed suggests that, for the moment, it is not a significant virulence mechanism in the analyzed region.

In recent years, biofilm formation by *Pseudomonas aeruginosa* has been observed with a prevalence ranging from 40% to 90%. The strains were categorized based on biofilm production ability, with strong producers comprising 33–47%, moderate producers 26–45%, and weak producers 21–33% of strains, respectively [72,73,74,75,76,77]. In our experiment, we showed that among 98 strains, as many as 97 of them showed biofilm-forming ability. After both 48 h and 72 h strong producers predominated among them, followed by medium producers, and only a small group had weak or no biofilm-forming ability. Our results are consistent with those obtained by Alonso et al. (2020). For their strains, 84.4% were strong, 14.5% were medium, and 1.1% were weak biofilm producers [78]. This poses a problem in veterinary medicine because it is difficult to get rid of the biofilm structure from the surfaces of production halls and veterinary clinics, so it can lead to the persistence of sources of infection and spread to more animals. For strains capable of producing this structure, treatment with antibiotics may be hindered due to reduced penetration into the depths [79]. As a result, this can lead to persistent and recurrent infections in patients, thereby causing prolonged inflammation, leading to tissue damage that can manifest as worsening of symptoms at relapse [80]. That is why prevention and monitoring of *P. aeruginosa* infections are so important to prevent the spread of strains that are strong biofilm producers. In treatment, it is important to use drugs to which the bacteria are sensitive and use effective cleaning and disinfection procedures in the facilities.

We tested the ability to produce biofilm at both phenotypic and genotypic levels. We noted that the frequency of genes responsible for factors involved in biofilm formation (*pel*A, *end*A, *alg*D, and *opr*F) do not always correlate with the strength of the biofilm they form.

Expression of *alg*D leads to the production of an enzyme involved in the synthesis of an exopolysaccharide, i.e., alginate, which protects bacterial cells from unfavorable environmental conditions and increases their adhesion to the surface. This is one of the elements of the biofilm whose production increases after attachment to the surface [81]. Among our strains, it appeared in 85% of samples, while in Rajabi et al.’s 2022 study, it was determined at 78.6%. Additionally, according to their study, the presence of the *alg*D gene increases biofilm production among *P. aeruginosa* strains [82], while our study does not confirm such a correlation, as it occurred with similar frequency after 48 and 72 h in both strong (87%; 82%), medium (78%; 89%) and weak (100%; 100%) producers.

The *pel*A gene is present in all strains included in the study (100%). It is responsible for encoding a protein with a polysaccharide deacetylase domain that is involved in the formation of the polysaccharide Pel, which is an intercellular adhesin involved in biofilm formation. For the production of this polysaccharide, products of the seven-gene *pel*A-*pel*G operon are required for strains to be able to produce a Pel-dependent biofilm and exhibit a Pel-associated phenotype [83]. Since the *pel*A gene was present in all our strains, regardless of the type of biofilm they formed, this leads us to believe that its presence does not affect the strength of the biofilm formed. The same conclusion was reached by Płókarz et al. in 2022 in their study, where the *pel*A gene was determined in only 38.7% of the strains, and no correlation was found between this gene and biofilm formation [77]. Due to the high frequency of this gene, we believe that a reasonable alternative treatment option is to use bacterial exopolysaccharide biosynthetic glycoside hydrolases along with colistin for clinical *P. aeruginosa* infections in our region because they increase the antimicrobial activity of the antibiotic without adversely affecting mammalian eukaryotic cells [84]. Since colistin is used in the treatment of livestock like cattle and poultry, where targeted therapies are not always used, this would allow the use of lower doses of the active substance and reduce the introduction of colistin into the environment, which could result in a reduction in the emergence of resistance to the drug among other bacteria. Baker P. et al. (2016) showed that treatment of PelAh-dependent *P. aeruginosa* biofilms increased the rate of microbial killing by neutrophils by about twice [84].

The *end*A gene encodes an endonuclease (DNA-specific endonuclease I), an enzyme responsible for degrading eDNA during biofilm dispersion, a process that allows bacteria to detach from the biofilm structure and colonize new surfaces. According to a study by Cherny et al. (2019), the presence of the *end*A gene does not result in a significant reduction in biofilm biomass, but stimulation of its expression significantly reduces the biomass of biofilms formed [85]. Our results also suggest that there is no correlation between the presence of this gene and the strength of the biofilm produced among the strains analyzed. Due to the frequent occurrence among our strains of the *end*A gene, based on research by Gnanadhas et al. (2015), in order to improve the efficacy of *P. aeruginosa* infection therapy for lower respiratory tract infection, it seems reasonable to consider the addition of a low concentration (0.5 µL) of L-methionine to antibiotic treatment. It has been shown in a mouse model that such a treatment improves the survival rate of patients, by stimulating the expression of endonucleases and thus degrading the biofilm structure in the lungs and releasing bacteria from it, which thus become more susceptible to antibiotics [48].

The *Opr*F gene is responsible for the presence in the outer cell membrane of the bacteria of a major pore affecting virulence in *P. aeruginosa*. Its absence results in reduced cell adhesion, secretion of toxins and signaling molecules. We determined this gene in 79% of our samples. In a study from Bukhari et al. (2020) it was shown that the presence of the *opr*F gene affects the rate of biofilm formation; mutants lacking *opr*F showed slower growth [86]. Our strains showed no such relationship, which suggests the absence of mutations within this gene in our strains. Nevertheless, the presence of this gene among the pathogens analyzed by us is good information because it gives hope for the use of protective vaccination in animals in our region [87]. Vaccines based on the *opr*L and *opr*F genes have been successfully used on chickens, where a dose of 100 µg of divalent vaccine stimulated both humoral and cellular responses protecting the animals from infection [88]. It also was tested on a mouse model during chronic respiratory infections, where two different immunization methods involving threefold administration of the vaccine to the animals were used. A strategy using two inoculations of i.d. pVR1020/oprF DNA and one with a chimeric virus with an *opr*F protective epitope on its surface yielded twice as good results as threefold immunization with a vaccine with plasmid DNA encoding the *opr*F/I infusion protein, resulting in 60.7% of infected mice having their infection-induced lesions completely resolved [89]. Moreover, in other studies where the efficacy of subcutaneous immunization with *opr*F and *opr*I with or without flagellin B was tested, the production of antibodies effective against mucosal as well as non-mucosal strains of *P. aeruginosa* was proven. Immunized mice showed significantly prolonged survival time, less bacteremia and fewer lung lesions during infection [90]. In studies using a variety of sera, it was observed that after one year, a single immunization of previously vaccinated patients allowed them to continue to induce a strong specific immune response [91].

The *gac*A gene, which occurs at a frequency of 86%, is responsible for encoding a response regulator of gram negative bacteria. It regulates the production of exoenzymes and secondary metabolites in *Pseudomonas* spp. Parkinsa et al. (2001) demonstrated that *P. aeruginosa* strains lacking the *gac*A gene showed a tenfold lower biofilm forming capacity [92]. In the case of our strains, even in the absence of this gene, a strong biofilm was still observed.

All the strains in our studies taken from dog genito-urinary tracts (*n* = 12) harbored all four genes responsible for biofilm production, and 10 of them had the *gac*A gene, indirectly affecting its production. They were able to produce biofilm formation with strong (*n* = 9) and medium (*n* = 3) intensity after 48 h and 72 h. They also showed 100% presence of the *exo*T and *plc*H genes, whose expression products are responsible for destroying epithelial cells, which may suggest that this is a hallmark of pathogenesis for these infections. These isolates also harbored the *exo*U gene (42%), which, as reported by Płókarz et al. (2023), may suggest increased resistance to marbofloxacin and amikacin [93]. In our study, this gene was more than twice as prevalent, so this may suggest a larger scale of the problem in treating infections in dogs. But this requires further study because it may be due to the difference in the number of strains tested in these two cases.

A better understanding of the functioning of the biofilm structure, as well as of the mechanisms determining antibiotic resistance and resistance to the bactericides associated with it, is necessary for the development of effective strategies for the prevention and control of biofilm-associated infections. Knowledge of virulence factors and their frequency of occurrence allows the development of methods of alternative to antibiotics for the treatment of bacterial infections, through inhibition of the synthesis of compounds both necessary for their growth and those responsible for virulence.

Genotyping of *P. aeruginosa* strains is extremely important to control the epidemiological situation and the appearance of new strains in the environment. Thus, it is possible to obtain information about the appearance of new outbreaks of infection and the direction of their spread. For this purpose, the use of the ERIC-PCR method is very well suited due to its speed and ease of application [94]. Targeting highly conserved repetitive sequence elements known as ERIC sequences common to Gram-negative enteric bacteria allows clear differentiation between different bacterial strains among *P. aeruginosa* species [95].

Interpretable ERIC pattern was obtained for all DNA isolates of tested strains, and using this method was a good approach for molecular typing of *P. aeruginosa* strains examined in this study. Based on electropherograms obtained after strain differentiation by ERIC-PCR, the profiles of DNA bands were compared for all tested *P. aeruginosa* strains. For this purpose, bioinformatics software was used—BioNumerics, which has a number of statistical tools enabling clustering of results and their analysis. The result of the study was to obtain dendrograms with the described similarity of the strains expressed as a percentage, which facilitates the comparison of strains based on the analysis of clusters, which include isolates with a similar ERIC pattern. BioNumerics enables the determination of the degree of epidemiological relatedness of these strains. After analyzing the ERIC-PCR products, 87 different ERIC profiles were obtained for our collection of 98 strains. Conversely, among 67 human clinical isolates described by Hematzadeh and Haghkhah (2021), only 38 divergent ERIC fingerprints were obtained. This could be related to the time and place of isolation as these strains were isolated in Iran between January 2016 and February 2017, or just with the fact that those were human isolates [95]. However, these observations are consistent with those described by Brzozowski et al. (2020), whose study of strains collected from humans in hospitals in Poland in 2020 showed that out of 202 isolates, only 30 related groups were obtained [96]. However, here, an additional factor could play a role as Brzozowski et al. used the PFGE technique for genotyping, which can have different discriminatory power than ERIC-PCR [97,98]. Such a high number of profiles in our study indicates that the strains affecting the animals are more genetically diverse, which may pose a problem for the development of a single effective treatment therapy and prevention strategy.

## 5. Conclusions

The results we obtained are worrying as they show high genetic variability among *P. aeruginosa* isolates, their high capacity for biofilm production at both phenotypic and genotypic levels, and the emergence of mechanisms that may cause a more severe disease course in animals in the future. Therefore, the virulence and resistance mechanisms of this pathogen should be continuously monitored by different research teams. In order to assess the scale of the risk posed by the strains we analyzed, drug resistance tests still need to be carried out. However, these results already demonstrate the severity of the problem for both human and veterinary medicine due to the high capacity of this pathogen to form a biofilm and thus increased resistance to antibiotics. In our opinion, preventive and curative measures should be directed towards the biofilm structure, as well as to raising the awareness of veterinarians and owners on the appropriate response and choice of therapy in case of confirmation of this bacterium in animals.

## Figures and Tables

**Table 1:** Characteristics of the collection sites of *P. aeruginosa* strains isolated from animals.

Characteristic	Overall (*n* = 98)	Dogs (*n* = 65)	Cats (*n* = 16)	Fowl (*n* = 10)	Ruminants (*n* = 7)
External auditory canal	17	16	1	-	-
Respiratory system					
Nasal cavity	22	7	15	-	-
Larynx	1	1	-	-	-
Trachea and bronchi	2	2	-	-	-
Conjunctival sac	10	10	-	-	-
Skin	15	15	-	-	-
Vagina	9	9	-	-	-
Urine	3	3	-	-	-
Perianal sinus glands	2	2	-	-	-
Goiter/cloaca	10	-	-	10	-
Milk	7	-	-	-	7

**Table 2:** Percentage of genes present by animal species.

Gene/Species	Dogs (*n* = 65) [*n*/%]	Cats (*n* = 16) [*n*/%]	Ruminants (*n* = 7) [*n*/%]	Fowl (*n* = 10) [*n*/%]
*tox*A	29 (45%)	9 (56%)	3 (43%)	3 (30%)
*exo*U	22 (34%)	8 (50%)	2 (29%)	3 (30%)
*exo*T	52 (80%)	14 (88%)	5 (71%)	9 (90%)
*exo*S	31 (48%)	8 (50%)	3 (43%)	6 (60%)
*las*B	60 (92%)	16 (100%)	7 (100%)	10 (100%)
*plc*N	30 (46%)	8 (50%)	3 (43%)	6 (60%)
*plc*H	56 (86%)	15 (94%)	6 (86%)	7 (70%)
*pld*A	17 (26%)	2 (13%)	3 (43%)	2 (20%)
*apr*A	55 (85%)	13 (81%)	6 (86%)	8 (80%)
*gac*A	53 (82%)	15 (94%)	7 (100%)	9 (90%)
*alg*D	53 (82%)	14 (88%)	7 (100%)	9 (90%)
*pel*A	65 (100%)	16 (100%)	7 (100%)	10 (100%)
*end*A	62 (95%)	15 (94%)	7 (100%)	10 (100%)
*opr*F	48 (74%)	15 (94%)	6 (86%)	8 (80%)

**Table 3:** Detection of biofilm formation.

Type of Biofilm Producer	Number of Isolates After	
	48 h [*n*/%]	72 h [*n*/%]
Strong	75 (77%)	74 (76%)
Moderate	18 (18%)	19 (19%)
Weak	4 (4%)	4 (4%)
None	1 (1%)	1 (1%)

**Table 4:** Percentage prevalence of biofilm-responsive genes among strong, medium, and weak producers after 48 and 72 h.

Gene/Biofilm Growth	Strong		Medium		Weak	
	48 h (*n* = 75) [*n*/%]	72 h (*n* = 74) [*n*/%]	48 h (*n* = 18) [*n*/%]	72 h (*n* = 19) [*n*/%]	48 h (*n* = 4) [*n*/%]	72 h (*n* = 4) [*n*/%]
*pel*A	75 (100%)	74 (100%)	18 (100%)	19 (100%)	4 (100%)	4 (100%)
*alg*D	65 (87%)	61 (82%)	14 (78%)	16 (84%)	4 (100%)	4 (100%)
*gac*A	66 (88%)	65 (87%)	14 (78%)	15 (79%)	3 (75%)	3 (75%)
*end*A	71 (95%)	71 (96%)	17 (94%)	18 (95%)	3 (75%)	3 (75%)
*opr*F	60 (80%)	59 (80%)	13 (72%)	14 (74%)	3 (75%)	3 (75%)

## Data Availability

All data generated or analyzed during this study are available from the corresponding author on reasonable request.

## Supplementary Materials

The following supporting information can be downloaded at: https://www.mdpi.com/article/10.3390/pathogens13110979/s1, Figure S1: A dendrogram of ERIC-PCR profiles of *Pseudomonas aeruginosa* strains.; Table S1: Characteristics of *Pseudomonas aeruginosa* strains considered in the study. Species and sampling locations, gene sets and ERIC-PCR profiles; Table S2: Primer sets used for virulence and biofilm capacity factors in *Pseudomonas aeruginosa* strains isolated from clinical cases in Poland.

## Abbreviations

QS—quorum sensing, PCR—polymerase chain reaction, EPS—extracellular polymeric substances, AHLs—acyl-homoserine lactones, T—time, U—voltage, TSB—tryptic soy broth.
