# Supplementary material for: Does Every Strain of Pseudomonas aeruginosa Attack the Same? Results of a Study of the Prevalence of Virulence Factors of Strains Obtained from Different Animal Species in Northeastern Poland
Source: Pathogens. 2024 Nov 8;13(11):979. doi: 10.3390/pathogens13110979 (PMC11597259; doi:10.3390/pathogens13110979)
Supplement: Supplementary file 1 [file pathogens-13-00979-s001.zip › Table S1.pdf]

Table S1. Characteristics of *Pseudomonas aeruginosa* strains considered in the study. Species and sampling locations, gene sets and ERIC-PCR profiles.

**Number of sample ERIC-PCR**	**Sample**	**Genes**	**ERIC-PCR Profile**
18	Cat - nasal cavity	*toxA, exoS, lasB, aprA, gacA, algD, pelA, endA, oprF*	14
27	Cat - nasal cavity	*toxA, exoT, exoS, lasB, plcN, plcH, aprA, gacA, algD, pelA, endA, oprF*	82
33	Cat - nasal cavity	*exoU, exoT, exoS, lasB, plcN, plcH, aprA, algD, pelA, endA, oprF*	20
34	Cat - nasal cavity	*exoU, exoT, lasB, plcH, gacA, algD, pelA, endA, oprF*	22
37	Cat - nasal cavity	*toxA, exoT, exoS, lasB, plcN, plcH, aprA, gacA, pelA, endA, oprF*	23
39	Cat - nasal cavity	*toxA, exoT, exoS, lasB, plcH, aprA, gacA, algD, pelA, endA, oprF*	26
42	Cat - nasal cavity	*exoU, exoT, lasB, plcH, gacA, algD, pelA, endA, oprF*	27
45	Cat - nasal cavity	*exoU, exoT, lasB, plcH, aprA, gacA, algD, pelA, endA, oprF*	29
46	Cat - nasal cavity	*exoU, lasB, plcH, aprA, gacA, algD, pelA, endA, oprF*	31
52	Cat - nasal cavity	*exoU, exoT, lasB, plcH, aprA, gacA, algD, pelA, endA, oprF*	32
62	Cat - nasal cavity	*toxA, exoT, exoS, lasB, plcN, plcH, pldA, aprA, gacA, algD, pelA, endA, oprF*	32
69	Cat - nasal cavity	*toxA, exoT, lasB, plcN, plcH, aprA, gacA, algD, pelA, endA, oprF*	48
77	Cat - nasal cavity	*toxA, exoT, exoS, lasB, plcN, plcH, pldA, aprA, gacA, algD, pelA, endA, oprF*	51
85	Cat - nasal cavity	*toxA, exoU, exoT, lasB, plcH, aprA, gacA, algD, pelA, endA, oprF*	48
97	Cat - nasal cavity	*toxA, exoU, exoT, lasB, plcN, plcH, gacA, pelA, endA*	58
101	Cat - external auditory canal	*exoT, exoS, lasB, plcN, plcH, aprA, gacA, algD, pelA, oprF*	80
100	Dog – trachea and bronchi	*toxA, exoU, exoT, plcN, plcH, aprA, gacA, algD, pelA, endA, oprF*	2

28	Dog – trachea and bronchi	*toxA, exoU, exoT, lasB, plcH, aprA, gacA, algD, pelA, endA, oprF*	4
86	Dog – trachea and bronchi	*toxA, exoT, lasB, plcN, plcH, aprA, gacA, algD, pelA, endA, oprF*	8
78	Dog – larynx	*exoT, exoS, lasB, plcN, plcH, aprA, gacA, algD, pelA, endA, oprF*	9
10	Dog – nasal cavity	*toxA, exoT, lasB, plcH, pldA, aprA, gacA, pelA, endA, oprF*	10
35	Dog – nasal cavity	*toxA, exoS, lasB, plcN, plcH, aprA, gacA, algD, pelA, endA, oprF*	11
36	Dog – nasal cavity	*toxA, exoT, exoS, lasB, plcN, plcH, aprA, gacA, algD, pelA, endA, oprF*	13
41	Dog – nasal cavity	*exoT, lasB, plcH, aprA, gacA, algD, pelA, endA, oprF*	15
66	Dog – nasal cavity	*exoT, exoS, lasB, plcN, plcH, aprA, gacA, algD, pelA, endA*	16
73	Dog – nasal cavity	*exoT, lasB, plcN, aprA, gacA, algD, pelA, endA, oprF*	17
67	Dog – perianal sinus glands	*exoU, exoT, exoS, lasB, plcN, plcH, pldA, aprA, gacA, algD, pelA, endA, oprF*	32
70	Dog – perianal sinus glands	*lasB, plcN, plcH, aprA, gacA, algD, pelA, endA, oprF*	74
57	Dog – urine	*exoU, exoT, exoS, lasB, plcH, aprA, gacA, algD, pelA, endA, oprF*	24
81	Dog – urine	*exoU, exoT, exoS, lasB, plcN, plcH, pldA, aprA, gacA, algD, pelA, endA, oprF*	25
83	Dog – urine	*toxA, exoU, exoT, lasB, plcN, plcH, aprA, gacA, algD, pelA, endA, oprF*	28
54	Dog – conjuctival sac	*exoS, lasB, plcH, aprA, gacA, pelA, endA, oprF*	30

58	Dog – conjuctival sac	*toxA, exoU, exoT, exoS, lasB, plcH, aprA, gacA, algD, pelA, endA, oprF*	75
79	Dog – conjuctival sac	*toxA, exoT, exoS, lasB, plcN, plcH, aprA, gacA, algD, pelA, endA, oprF*	33
80	Dog – conjuctival sac	*toxA, exoT, exoS, lasB, plcH, aprA, gacA, algD, pelA, endA, oprF*	34
82	Dog – conjuctival sac	*exoT, exoS, lasB, plcN, plcH, aprA, gacA, algD, pelA, endA, oprF*	35
95	Dog – conjuctival sac	*toxA, exoT, lasB, plcH, pldA, aprA, gacA, algD, pelA, endA, oprF*	19
103	Dog – conjuctival sac	*exoU, exoT, lasB, plcN, plcH, pldA, aprA, gacA, pelA, endA*	41
105	Dog – conjuctival sac	*exoT, exoS, lasB, plcN, plcH, pldA, aprA, gacA, pelA, endA*	43
107	Dog – conjuctival sac	*toxA, exoU, exoT, lasB, plcN, plcH, pldA, gacA, pelA, endA*	44
94	Dog – conjuctival sac	*toxA, exoU, exoT, lasB, plcN, plcH, aprA, gacA, algD, pelA, endA, oprF*	45
17	Dog- vagina	*toxA, exoT, exoS, lasB, plcN, plcH, pldA, aprA, gacA, algD, pelA, endA, oprF*	46
50	Dog- vagina	*exoT, exoS, lasB, plcH, pldA, gacA, algD, pelA, endA, oprF*	49
68	Dog- vagina	*exoT, exoS, lasB, plcN, plcH, aprA, gacA, algD, pelA, endA, oprF*	5
76	Dog- vagina	*toxA, exoT, lasB, plcH, aprA, algD, pelA, endA, oprF*	76
88	Dog- vagina	*toxA, exoU, exoT, exoS, lasB, plcH, aprA, gacA, algD, pelA, endA, oprF*	48
89	Dog- vagina	*toxA, exoT, lasB, plcH, aprA, algD, pelA, endA, oprF*	50
90	Dog- vagina	*exoU, exoT, lasB, plcH, pldA, aprA, gacA, algD, pelA, endA, oprF*	77
110	Dog- vagina	*exoT, exoS, plcN, plcH, pldA, gacA, algD, pelA, endA, oprF*	51

9	Dog- vagina	*toxA, exoT, exoS, lasB, plcH, aprA, gacA, algD, pelA, endA, oprF*	51
109	Dog - skin	*toxA, exoT, lasB, plcN, plcH, gacA, pelA, endA*	48
106	Dog - skin	*plcN, algD, pelA*	53
7	Dog - skin	*exoU, exoT, lasB, plcN, plcH, gacA, pelA, endA*	54
20	Dog - skin	*exoT, exoS, lasB, aprA, gacA, algD, pelA, endA, oprF*	55
43	Dog - skin	*exoU, exoS, lasB, plcH, aprA, gacA, algD, pelA, endA*	56
61	Dog - skin	*exoU, exoT, lasB, plcH, aprA, gacA, algD, pelA, endA, oprF*	38
64	Dog - skin	*exoT, exoS, lasB, plcH, pldA, aprA, algD, pelA, endA, oprF*	55
65	Dog - skin	*lasB, plcN, aprA, pelA, endA*	37
71	Dog - skin	*toxA, exoT, exoS, lasB, plcH, aprA, gacA, algD, pelA, endA, oprF*	78
84	Dog - skin	*toxA, exoU, exoT, lasB, plcH, aprA, gacA, algD, pelA, endA, oprF*	59
99	Dog - skin	*lasB, plcN, plcH, aprA, gacA, pelA, oprF*	60
102	Dog - skin	*exoU, exoT, lasB, plcN, plcH, pldA, gacA, pelA, endA*	87
111	Dog - skin	*toxA, exoT, exoS, plcN, plcH, gacA, algD, pelA, endA*	61
44	Dog - skin	*toxA, lasB, plcH, aprA, gacA, algD, pelA, endA, oprF*	57
32	Dog - skin	*toxA, exoT, lasB, plcH, pldA, aprA, gacA, pelA, endA, oprF*	63
2	Dog – external auditory canal	*exoT, lasB, plcH, aprA, gacA, algD, pelA, endA, oprF*	79
13	Dog – external auditory canal	*toxA, exoU, exoT, exoS, lasB, plcH, aprA, algD, pelA, endA*	47
14	Dog – external auditory canal	*exoT, lasB, pelA, endA*	64
15	Dog – external auditory canal	*exoT, lasB, plcH, aprA, algD, pelA, endA, oprF*	66
22	Dog – external auditory canal	*lasB, plcH, pldA, aprA, gacA, algD, pelA, endA, oprF*	81

24	Dog – external auditory canal	*toxA, exoT, exoS, lasB, aprA, gacA, algD, pelA, endA*	67
29	Dog – external auditory canal	*toxA, exoT, exoS, lasB, plcN, aprA, algD, pelA, endA*	68
47	Dog – external auditory canal	*exoS, lasB, plcH, pldA, aprA, gacA, algD, pelA, endA, oprF*	83
48	Dog – external auditory canal	*exoS, lasB, plcH, aprA, gacA, algD, pelA, oprF*	84
55	Dog – external auditory canal	*exoT, exoS, lasB, plcH, aprA, algD, pelA, endA, oprF*	70
60	Dog – external auditory canal	*exoU, exoS, lasB, plcH, aprA, algD, pelA, endA, oprF*	56
63	Dog – external auditory canal	*toxA, exoU, exoT, lasB, plcN, plcH, aprA, gacA, algD, pelA, endA*	71
87	Dog – external auditory canal	*exoU, exoT, lasB, pldA, aprA, gacA, algD, pelA, endA, oprF*	85
92	Dog – external auditory canal	*exoU, exoT, lasB, plcH, aprA, gacA, algD, pelA, endA, oprF*	86
93	Dog – external auditory canal	*toxA, exoT, lasB, plcH, aprA, gacA, algD, pelA, endA, oprF*	7
108	Dog – external auditory canal	*plcN, algD, pelA, endA*	21
1	Fowl – goiter/cloaca	*exoU, exoT, exoS, lasB, plcN, pldA, gacA, algD, pelA, endA, oprF*	1
5	Fowl – goiter/cloaca	*exoU, exoT, exoS, lasB, plcN, plcH, aprA, gacA, algD, pelA, endA*	39
16	Fowl – goiter/cloaca	*exoT, exoS, lasB, plcH, aprA, gacA, algD, pelA, endA, oprF*	12

31	Fowl – goiter/cloaca	*exoT, lasB, aprA, algD, pelA, endA, oprF*	21
38	Fowl – goiter/cloaca	*toxA, exoT, lasB, plcN, aprA, gacA, algD, pelA, endA, oprF*	74
51	Fowl – goiter/cloaca	*exoT, exoS, lasB, plcH, aprA, gacA, algD, pelA, endA, oprF*	36
53	Fowl – goiter/cloaca	*exoT, lasB, plcH, aprA, gacA, algD, pelA, endA, oprF*	40
56	Fowl – goiter/cloaca	*exoU, lasB, plcN, plcH, aprA, gacA, algD, pelA, endA, oprF*	42
96	Fowl – goiter/cloaca	*toxA, exoT, exoS, lasB, plcN, plcH, pldA, aprA, gacA, algD, pelA, endA, oprF*	65
104	Fowl – goiter/cloaca	*toxA, exoT, exoS, lasB, plcN, plcH, gacA, pelA, endA*	69
3	Ruminant - milk	*exoU, exoT, lasB, plcN, aprA, gacA, algD, pelA, endA, oprF*	3
6	Ruminant - milk	*toxA, lasB, plcH, aprA, gacA, algD, pelA, endA, oprF*	4
8	Ruminant - milk	*exoU, exoT, exoS, lasB, plcN, plcH, pldA, aprA, gacA, algD, pelA, endA, oprF*	6
25	Ruminant - milk	*exoT, lasB, plcH, pldA, aprA, gacA, algD, pelA, endA*	73
26	Ruminant - milk	*toxA, exoT, lasB, plcH, aprA, gacA, algD, pelA, endA, oprF*	18
72	Ruminant - milk	*exoT, exoS, lasB, plcH, gacA, algD, pelA, endA, oprF*	52
98	Ruminant - milk	*toxA, exoS, lasB, plcN, plcH, pldA, aprA, gacA, algD, pelA, endA, oprF*	66
