# Supplementary material for: Does Every Strain of Pseudomonas aeruginosa Attack the Same? Results of a Study of the Prevalence of Virulence Factors of Strains Obtained from Different Animal Species in Northeastern Poland
Source: Pathogens. 2024 Nov 8;13(11):979. doi: 10.3390/pathogens13110979 (PMC11597259; doi:10.3390/pathogens13110979)
Supplement: Supplementary file 1 [file pathogens-13-00979-s001.zip › Table S2.pdf]

Table S2. Primer sets used for virulence and biofilm capacity factors in *Pseudomonas aeruginosa* strains isolated from clinical cases in Poland.

Gene Name	Encoded Virulence Factor	Function	Primer Sequence	Melting Temperature (°C)	Annealing Temperature (°C)	Product size	Reference
*tox*A	Exotoxin A	Protein synthesis inhibition	F: 5′ GGTAACCAGCTCAGCCACAT 3′	53,8	50	352 bp	[10]
			R: 5′ TGATGTCCAGGTCATGCTTC 3′	51,8			
*exo*U	Exotoxin U	Anti-phagocytosis	F: 5′ CTCAATGTACTCCCACGCATAG 3′	54,8	52	406 bp	[41]
			R: 5′ CATCCTGGAATTCTGTCCACTC 3′	54,8			
*exo*T	Exotoxin T	Interference with host cellular immunity	F: 5′ GCCGAGATCAAGCAGATGAT 3′	51,8	55	1155 bp	
			R: 5′ GACAGGCTCGCCCTTTAC 3′	52,6			
*exo*S	Exotoxin S	Anti-phagocytosis	F: 5′ CTTGAAGGGACTCGACAAGG 3′	53,8	50	504 bp	[42]
			R: 5′ TTCAGGTCCGCGTAGTGAAT 3′	51,8			
*las*B	Elastase B	Damaging tissue components	F: 5′ GGGAATGAACGAAGCGTTCTC 3′	51,8	49	300 bp	[43]
				55,9			

			R: 5′ GGTCCAGTAGTAGCGGTTGG 3′				
*plc*N	Phospholipase C (N)	Damaging tissue components	F: 5′ GTTATCGCAACCAGCCCTAC 3′	53,8	51	466 bp	[43]
			R : 5′ AGGTCGAACACCTGGAACAC 3′	53,8			
*plc*H	Phospholipase C (H)	Hemolysis	F: 5′ GAAGCCATGGGCTACTTCAA 3′	51,8	50	307 bp	[43]
			R: 5′ AGAGTGACGAGGAGCGGTAG 3′	55,9			
*pld*A	Phospholipase D	Antimicrobial activity	F: 5′ TGTCGATCACCACGGATTTC 3′	51,8	50	151 bp	[44]
			R: 5’ TATGACTTCGAAACCATGCTCG 3′	53			
*apr*A	Alkaline protease	Degradation of proteins, interference with the immune system	F: 5′ CAGACCCTGACCCACGAGAT 3′	55,9	47	445 bp	[45]
			R: 5′ CATTGCCCTTCAACCCG 3′	49,5			
*gac*A	Response regulator GacA	Regulation of virulence expression	F: 5′ CCAGATCGCCCTGATGATCGC 3′	58,3	51	108 bp	[46]
			R: 5′ TTCTCGAAGATGCGGTAGCG 3′	53,8			
*alg*D	GDPmannose	Adherence to surfaces	F: 5′ ATGCGAATCAGCATCTTTGGT 3′	50,5	48	1310 bp	[47]

	dehydrogenase		R: 5′ CTACCAGCAGATGCCCTCGGC 3′	60,2			
*pel*A	Pel polysaccharide	Intercellular adhesion	F: 5′ CCTTCAGCCATCCGT-TCTTCT 3′	53,8	51	118 bp	[48]
			R: 5′ TCGCGTACGAA-GTCGACCT 3′	54,4			
*end*A	DNA-specific endonuclease I	Biofilm spreading	F: 5′ GTTTGTAGGCCTTTTCGCCC 3′	53,8	50	237 bp	[49]
			R: 5′ GTAGAGCTTCCAGCCGATT 3′	53,8			
*opr*F	Major porin and structural outer membrane porin OprF precursor	Transmembrane transfer	F: 5′ TTAAAAGCTT ATGAAACTGAAGAACACCTTAG 3′	56,7	58	1053 bp	[50]
			R: 5′ TATA CTCGAGTTACTTGGCTTCRGCTTCT 3′	60,1			
