# Supplementary figures and images for: Does Every Strain of Pseudomonas aeruginosa Attack the Same? Results of a Study of the Prevalence of Virulence Factors of Strains Obtained from Different Animal Species in Northeastern Poland
Source: Pathogens. 2024 Nov 8;13(11):979. doi: 10.3390/pathogens13110979 (PMC11597259; doi:10.3390/pathogens13110979)

Figure S1. A dendrogram of ERIC-PCR profiles of *Pseudomonas aeruginosa* strains.

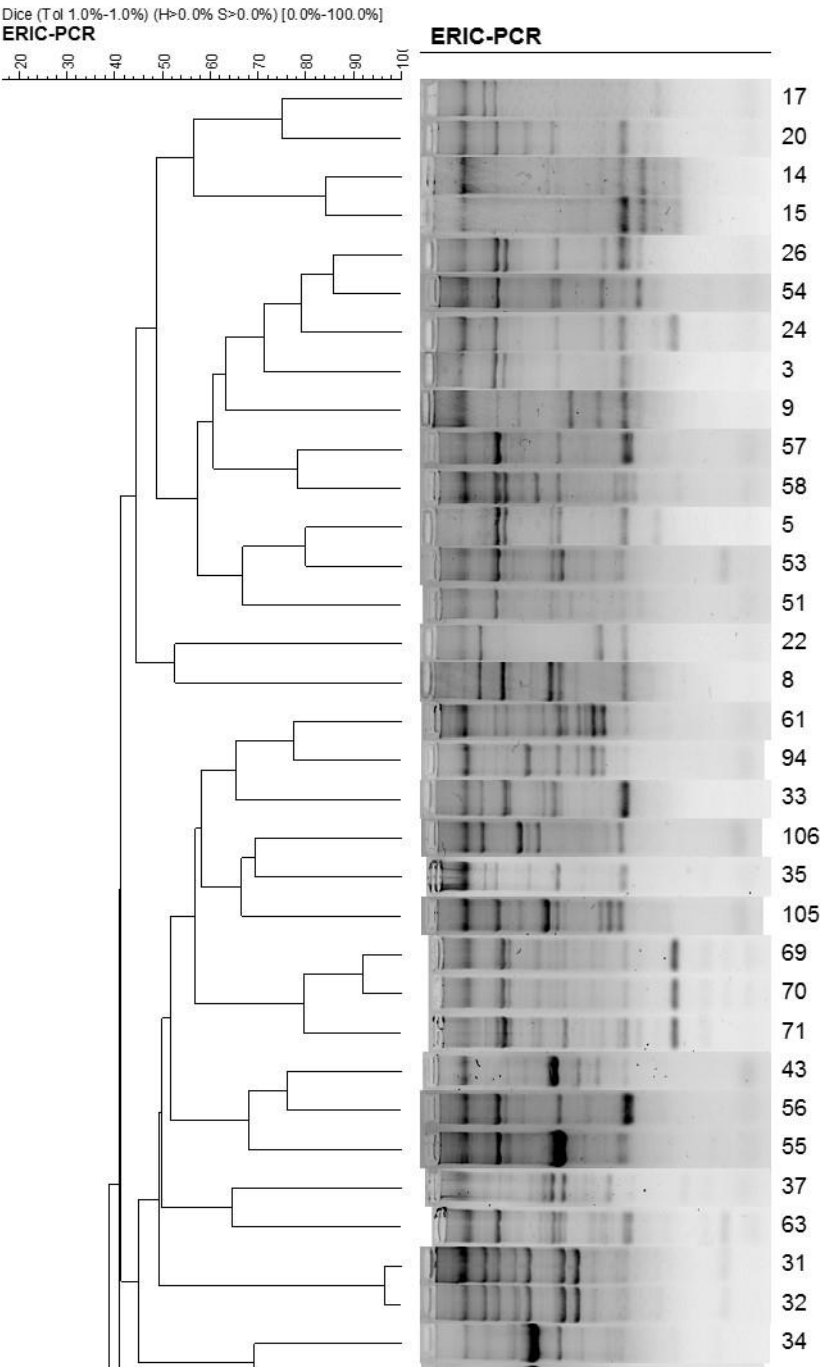

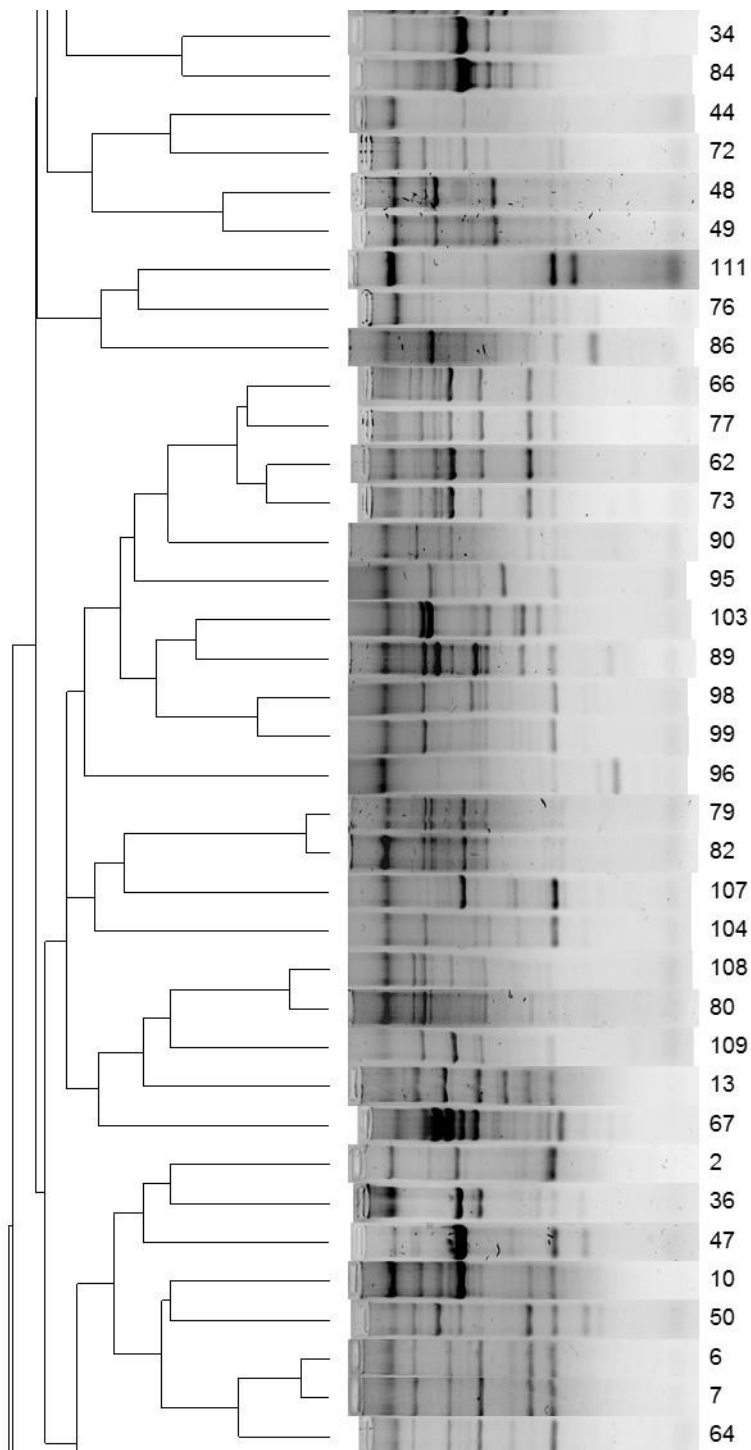

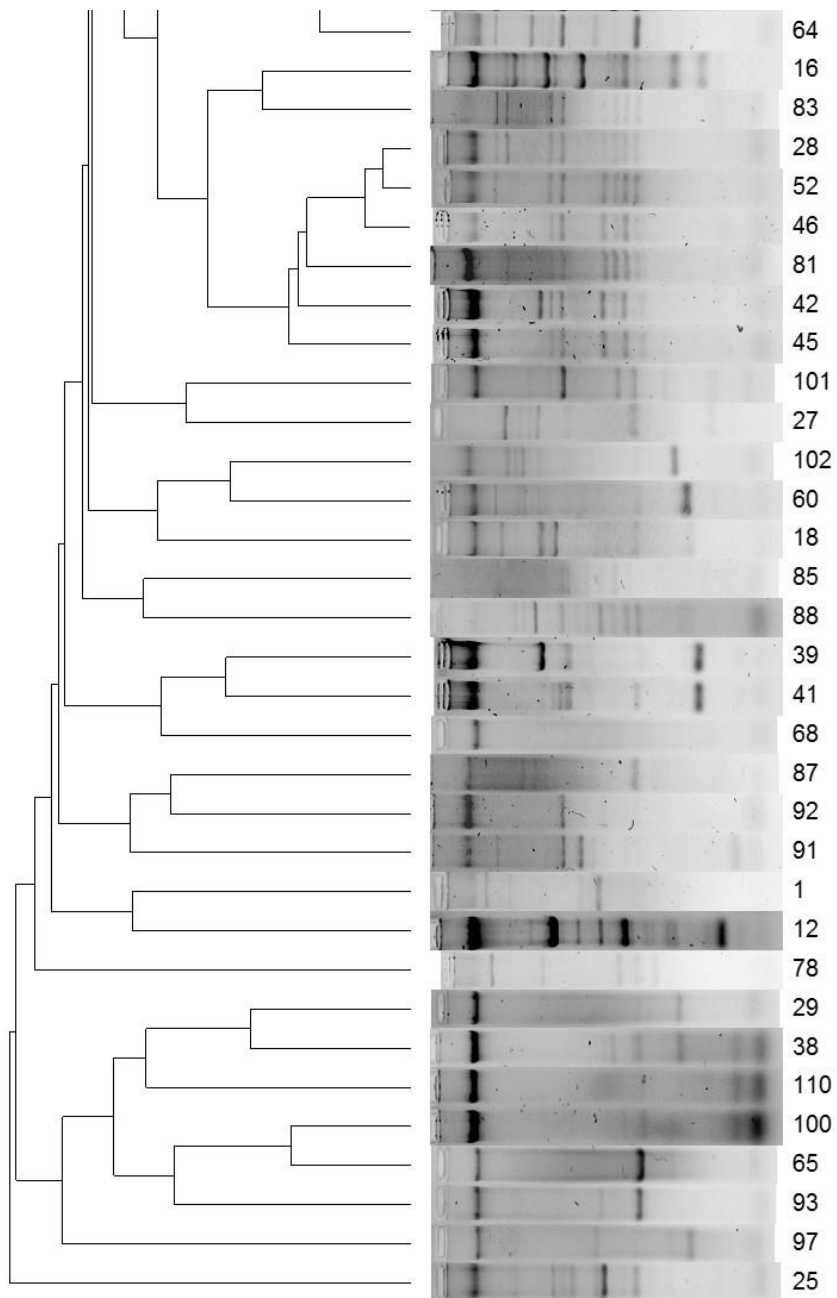

Supplement: Supplementary file 1 [file pathogens-13-00979-s001.zip › Figure S1.pdf]
